# Dentistry as a Profession

**Published:** 1862-05

**Authors:** 


### Selections.

---

### DENTISTRY AS A PROFESSION.

Comparatively few years have elapsed since dentistry has been elevated to a distinct profession in this country. From a very remote period, however, the dental art was practiced by a particular class. Among the Egyptians there were individuals, as history informs us, who practiced "stopping teeth with gold," after they had studied those precepts which had been laid down by their predecessors, and who made artificial teeth. Æsculapius III., the son of Arsippus and Arsinœ, is said to have been the first who extracted teeth. Hippocrates also gives instructions in regard to the time when and circumstances under which teeth should be cauterized. But their knowledge of the structure and development of the teeth was very limited, and their views extremely fanciful. Hippocrates describes the tooth as a gelatinous substance growing from the head and jaws, the fatty part of which is dried by heat and burned up. Aristotle asserts "that the teeth continue to grow and increase in length during life," and "that man has more teeth than woman." Aretæus considers the cause of toothache is known only to God. Scribonius asserts that loose teeth may be tightened by a decoc tion of patience-root in wine or ass's milk; and he mentions a great variety of tooth-powders, among which one composed of "burnt hartshorne, mastic, and salammoniac" may be mentioned. Marcellus recommends the use of amulets for the cure of the toothache, which seems to have been one of the prominent affections of the teeth recognized in days of antiquity. Galen, who appears to have had the best knowledge of this subject, gives a full description of the structure and formation of the teeth, and describes the various operations in dental surgery then known.

But dentistry was not then a distinct profession. But few operations save that of extraction were performed, and these all by those who were trained up to the practice of the healing art. The knowledge existing was all drawn from the experience of those who preceded them, and partook, in all its details, of the fanciful notions which were prevalent in medicine. Little was known of physiology; the notions of pathology were crude and undigested, and the practical operations were in entire consonance with the fancies and superstitions of the dark age in which they lived. It was reserved to a later period and to another age to assign to dentistry its proper position. It is now recognized as a distinct branch of the healing art. It has its teachers, its schools, and its diplomas distinct from all other branches of medicine, yet so intimately interwoven with them, that while one may be a pathologist without being a dentist, yet no one can be a good dentist without being a pathologist; while one may be a surgeon without understanding all the minutiæ of dental operations, yet no one can be a good dentist, practically, without a thorough knowledge of surgery. The barber, as of old, may learn to extract a tooth well, as a mere mechanical act; but a barber would hardly be qualified to manufacture and insert a set of teeth.

While, therefore, dentistry is and should be recognized as a distinct branch of the medical profession, it should be as distinctly asserted that there is an intimate relation between the two, a mutual dependence one upon the other; such a dependence as will lead to the utmost cordiality and courtesy As the surgeon, so the dentist should be a thorough anatomist. The surgeon who has a vague and indefinite knowledge of the anatomical structure of the part is poorly qualified to amputate a limb, ligate an artery, or extirpate a tumor with success; so the dental surgeon is equally unfit to perform intelligently and successfully any of the numerous manipulations of his art without some minute anatomical knowledge. The structure of the tooth, of the bones which enter into the construction of the mouth, the location and relation of the nerves and blood-vessels which supply that portion of the system with nutrition and with nervous energy—especially the ramification of that pair of nerves the disease or exposure of which will subtract almost the entire sum of happiness from life—should not the dentist understand these well and fully? Will not his manipulations, skillful though they may appear to the unpracticed eye, be but the vagaries of ignorance without such knowledge?

So also the dentist should be a good physiologist. Not that he should necessarily be able to determine the function of every organ of the body, as the physician, but the physiological structure and functions more particularly of those organs which are concerned with the mouth, and which have more or less to do with every change which disease effects in that region—*these* he should fully understand in all their relations to each other, in all the functions they perform. And he should not be content with a merely superficial knowledge of them. The more entirely familiar he is with their physiology, the better will he be prepared to recognize and remedy any deviation from the natural standard.

The dentist should also be a good pathologist. Understanding the normal functions, he should also understand the abnormal conditions. Diseases which affect the buccal structure and organs must necessarily come under his observation every day of his practical life. He will be compelled to take cognizance of them. They will present themselves in every variety of aspect and condition. Perhaps there may be an abnormal composition of the fluids, the secretions of the salivary glands. There may be an accumulation of matter in the antrum of Highmore from some cause. The root of a tooth may take on morbid action, and ulceration be the consequence. The nerves may become diseased and give rise to odontalgia, neuralgia, or even results still more terrible and disastrous. The dentist should fully understand the exact pathological condition in all these affections, as well as many others connected therewith, which will obtrude themselves unbidden upon his notice, and demand attention at his hands. So also he should have some knowledge of therapeutics or the application of remedies to the cure of disease. Not that he should become a general practitioner of medicine. His therapeutic knowledge may be general, and yet should be sufficiently minute to enable him to determine correctly and accurately as to the cause of the disease he encounters, and the proper remedies to be employed. The dentist is not the mere automaton that is to go through a certain round of manual operations and manipulations—the machine which, once set in motion, will grind out and polish off a beautiful piece of mechanism. He ought to understand fully all his operations, and be a good mechanician; but he ought to be more than that. The automaton may extract a tooth, or plug a cavity, or produce a faultless specimen of mechanical art; the thinking, reasoning man should do more. Such a one should be able to give a reason for the faith that is in him, to adjust his mechanical productions nicely to anatomical and pathological conditions, so as to give the best prospect of success.

The dentist should also have some knowledge of chemistry. The composition of the fluids, which are secreted by the glands of the mouth and by the stomach, bears an important relation to diseases of the mouth, as well as other portions of the system. Nature has compounded these fluids in just the exact proportion necessary for the function they are designed to fulfill. The dentist should understand these proportions, and be able to detect any deviation from them. The action of these fluids has an important bearing not only upon the structures themselves, but also upon the material used by the dentist in repairing the ravages of disease. Hence the necessity that he understand the chemical relations that exist between them. Without such knowledge, to his utter mortification and disgrace, his "gold may become dimmed, and his fine gold may become tarnished." The importance, therefore, of thorough education in all these particulars to the dentist who desires to occupy a distinguished place in his profession, is evident. The time has passed when dentistry is to be practiced by the ignoramus. The demands of the age require education in this branch of the medical profession as well as that which relates more especially to disease of the general system. The progress of events, the growing importance of the art, the rapid strides of mechanical improvement, have elevated dentistry to the position of a profession, and imperiously demand that the recipients of its honors shall be men of science as well as art, theoretical as practical; that they shall not be content to travel in one beaten track, like the horse in the tread-mill; that their ideas shall not run on perpetually in the same rut channeled out for them by the continued attrition of ages, and which will necessarily set them completely adrift when any new combinations arise; but that they shall be rooted and grounded in the fundamental principles of medicine generally, and so thoroughly versed in the science of their own particular speciality as that the medical profession will be pleased to recognize them as worthy members of the great fraternity, and will be compelled to award them the deference due to superior attainments in one important department.

Among dentists themselves also as members of a profession rapidly rising in importance, there ought to be an *esprit du corps* which shall lead them to be content only with the most rapid growth and progress in the art. To excel—not merely to reach a lazy mediocrity—to *excel* should be the motto. To elevate, not depress, the standard of attainment.

We are, ourselves, no dentists, but we offer the foregoing thoughts as a tribute to a specialty rapidly coming to the maturity of a learned and intelligent profession, and which, in its exalted position, as medical men, we most gladly recognize.—*Med. and Surg. Reporter.*

